# Worsening heart failure–based hierarchical endpoints beyond HF hospitalization: expert opinion paper

**DOI:** 10.1093/eschf/xvag107

**Published:** 2026-04-18

**Authors:** Agustín Fernández-Cisnal, Gema Miñana, Rafael de la Espriella, Enrique Santas, Joan Carles Trullas, Jan Biegus, Harriette Van Spall, Julio Núñez

**Affiliations:** Cardiology Department, Parc Sanitari Sant Joan de Deu, Hospital Sant Boi, Sant Boi de Llobregat, Barcelona, Spain; Cardiology Department, Hospital Clínico Universitario de València, Instituto de Investigación Sanitaria (INCLIVA), University of Valencia, Avenida Blasco Ibáñez 17, València 46010, Spain; Centro de Investigación Biomédica en Red Enfermedades Cardiovasculares (CIBERCV), C. de Melchor Fernández Almagro, 3, Fuencarral-El Pardo, 28029 Madrid, Spain; Cardiology Department, Hospital Clínico Universitario de València, Instituto de Investigación Sanitaria (INCLIVA), University of Valencia, Avenida Blasco Ibáñez 17, València 46010, Spain; Centro de Investigación Biomédica en Red Enfermedades Cardiovasculares (CIBERCV), C. de Melchor Fernández Almagro, 3, Fuencarral-El Pardo, 28029 Madrid, Spain; Cardiology Department, Hospital Clínico Universitario de València, Instituto de Investigación Sanitaria (INCLIVA), University of Valencia, Avenida Blasco Ibáñez 17, València 46010, Spain; Centro de Investigación Biomédica en Red Enfermedades Cardiovasculares (CIBERCV), C. de Melchor Fernández Almagro, 3, Fuencarral-El Pardo, 28029 Madrid, Spain; Internal Medicine Department, Hospital d’Olot i comarcal de la Garrotxa, Girona, Spain; Laboratori de Reparació i Regeneració Tissular (TR2Lab), Facultat de Medicina, Universitat de Vic-Universitat Central de Catalunya, Barcelona, Spain; Department of Cardiology, Clinical Department of Intensive Cardiac Care, Faculty of Medicine, Wroclaw Medical University, Institute of Heart Diseases, Borowska 213, Wroclaw 50-556, Poland; Department of Cardiology, Jan Mikulicz Radecki University Hospital in Wrocław, Borowska 213, Wroclaw 50-556, Poland; Department of Medicine, Faculty of Health Sciences, McMaster University, Population Health Research Institute, Hamilton, Canada; Cardiology Department, Hospital Clínico Universitario de València, Instituto de Investigación Sanitaria (INCLIVA), University of Valencia, Avenida Blasco Ibáñez 17, València 46010, Spain; Centro de Investigación Biomédica en Red Enfermedades Cardiovasculares (CIBERCV), C. de Melchor Fernández Almagro, 3, Fuencarral-El Pardo, 28029 Madrid, Spain

**Keywords:** Worsening heart failure, Clinical trial endpoints, Hierarchical composite, Total events

## Abstract

The traditional, hospitalization-centric composite endpoint of cardiovascular (CV) death or time-to-first heart failure (HF) hospitalization is increasingly misaligned with contemporary HF care and, as evidence-based therapies lower event rates over time, requires larger trials with longer follow-up. Improved survival, modern ambulatory pathways mean that a larger share of worsening HF is treated outside the hospital and that patients may experience recurrent worsening HF episodes. Relying on time-to-first hospitalization alone can therefore miss clinically relevant morbidity; recurrent-event approaches can offer additional power mainly when risk heterogeneity is high and treatment discontinuation after a first event is infrequent. To address this gap, we propose a standardized, adjudicated definition of worsening HF events informed by published consensus definitions, expanded to capture ambulatory events across care settings. Building on this definition, we recommend hierarchical primary endpoints that prioritize all-cause death with CV death evaluated as a secondary mortality outcome when prespecified and adjudicated, while robustly measuring morbidity through total adjudicated worsening HF events (first and recurrent), with validated patient-reported outcomes as additional hierarchical levels. We outline operational considerations for event capture and adjudication, including prioritized composite analytic approaches, and highlight safeguards to mitigate ascertainment bias and dilution by more subjectively defined events. Adoption of worsening HF -based hierarchical endpoints can better reflect the total disease burden, improve statistical power, and enhance interpretability across evolving care models.

## Graphical Abstract

**Graphical Abstract:**
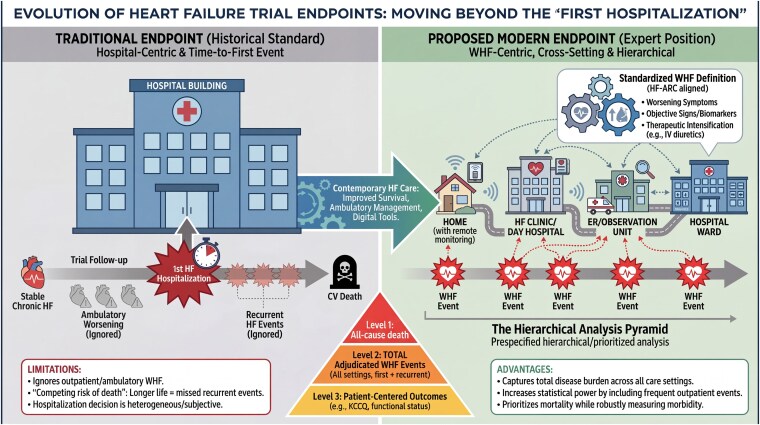
Beyond first hospitalization: a modern WHF centred endpoint framework. Framework spanning care settings (home, HF clinic/day hospital, ED/observation, inpatient ward) using a standardized WHF definition and hierarchical outcome assessment. Abbreviations: ED, emergency department; HF, heart failure; HF-ARC, Heart Failure Academic Research Consortium; IV, intravenous; WHF, worsening heart failure.

## Historical rationale for heart failure hospitalization as a trial endpoint

The composite endpoint of cardiovascular (CV) death or heart failure (HF) hospitalization emerged in the 1990s as a clinically compelling measure of treatment effect in chronic HF.^1^ At that time, HF was characterized by high short-term risk of mortality and HF admissions, limited outpatient infrastructure, and few disease-modifying therapies. Early large-scale trials of neurohormonal blockade showed that interventions which reduced HF admissions also improved survival, functional status, and quality of life.^2–6^ However, time to death or first HF hospitalization was reinforced as the primary endpoint reflecting the crucial points of the disease progression.

Subsequent trials such as CHARM (candesartan)^7^ and EMPHASIS-HF (eplerenone),^8^ or even more recent PARADIGM (sacubitril/valsartan),^9^ further reinforced the validity of this composite, showing concordant reductions in CV death and HF hospitalization together with fewer hospitalizations for worsening HF (WHF). For several years, this framework has proved suitable for this purpose. Accordingly, regulatory agencies and guideline-writing committees adopted CV death or HF hospitalization as a preferred primary endpoint, considering it clinically meaningful, objective enough to minimize the risk of ascertainment bias, and acceptable for regulatory decision-making.^10,11^

From a pathophysiologic perspective, this choice was also sensible. A first HF hospitalization usually signals a transition to a more advanced stage of disease, with sharply increased short-term mortality.^12^ The event is well documented, relatively objective, temporally discrete, and strongly linked to subsequent outcomes,^13,14^ so it provided an attractive and simple surrogate for disease progression.

**Figure 1:**
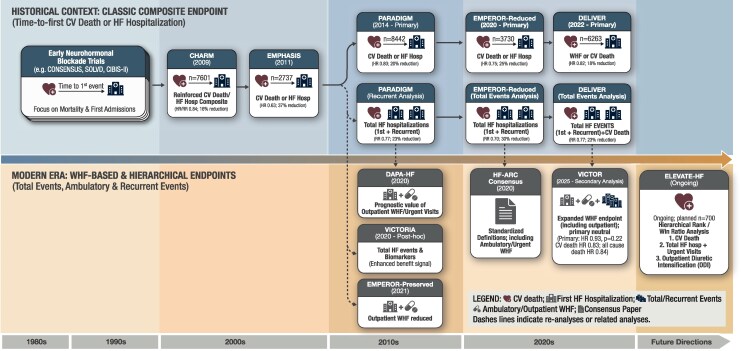
Evolution of heart failure trial endpoints. Shift from hospital centric time to first event endpoints to modern approaches capturing WHF, recurrent events, and hierarchical analyses. Abbreviations: CV, cardiovascular; HF, heart failure; WHF, worsening heart failure

In the current era, however, HF care, survival, and health system organization have changed substantially. Patients live longer with chronic HF, face recurrent worsening heart failure (WHF) events that in many cases are detected and treated in ambulatory settings (including clinician-directed oral diuretic intensification), and in some jurisdictions, can receive inpatient level care in outpatient settings.^15–18^ As a result, the historical time to composite event of CV death or first HF hospitalization seems progressively less well aligned with both the biology of chronic HF and the realities of modern care delivery.^15,17–20^ This evolution in trial endpoints is summarized in *Figure 1*. Thus, the event/endpoint in question has evolved from HF ‘pure’ hospitalization to a much broader entity: worsening of HF^19^ The latter one includes HF hospitalization as well as other more subjectively defined events, like unplanned hospitalization in the emergency department or escalation of loop diuretics that occur as a result of worsening symptoms and signs of HF.^19^ The potential advantages and limitations of adopting a broader WHF definition are summarized in *Table 1*.

**Table 1:** Pros and cons of a broader definition of worsening heart failure (WHF)

Potential advantages	Potential limitations
**Higher statistical power** A broader WHF definition increases event rates, thereby improving statistical efficiency and enhancing the ability to detect treatment effects.	**Pathophysiological coherence required** All components of the WHF definition should be mechanistically linked to HF progression. Events included solely for statistical reasons, without clear biological plausibility, may dilute interpretability.
**Smaller required sample size** With higher event rates, trials can be adequately powered with fewer participants, reducing costs and improving feasibility, particularly relevant in phase II studies or mechanistic trials.	**Prognostic validity** Each component should independently associate with adverse clinical outcomes. If a component lacks prognostic significance, the composite risks incorporate clinically trivial events.
**Lower risk of type II error** By capturing more events, the probability of falsely concluding no treatment effect is reduced.	**Clinical meaningfulness** Endpoints must reflect outcomes and the effect size that matter to patients (symptoms, functional capacity, survival). Administrative or minor therapeutic adjustments may not meet this threshold.
**Earlier signal detection** Broader WHF definitions may allow earlier identification of therapeutic signals, which is particularly useful in short-term or adaptive trial designs.	**Subjectivity and risk of bias** Some components may contain subjective elements and be susceptible to investigator bias, particularly in open-label trials.
**Operational feasibility** Broader criteria (e.g. intensification of diuretics) may be easier to capture than hard endpoints such as mortality or HF hospitalization, especially in pragmatic or registry-based trials.	**Surrogate endpoint limitations** Surrogate markers are often weakly or moderately correlated with treatment effects on all-cause mortality. There is insufficient evidence to support the use of a single biomarker or echocardiographic parameter as a standalone regulatory endpoint in HF trials.
	**Event heterogeneity and signal dilution** A heterogeneous composite may combine events of different severity and prognostic weight, potentially obscuring true effects on hard outcomes.
	**Regulatory interpretability** Regulatory agencies typically prioritize endpoints with established clinical validity. Broad definitions that rely heavily on surrogate or process measures may face scrutiny.

## Limitations of a time-to-first, hospitalization-centric endpoint in contemporary heart failure

### Competing risks and cumulative WHF morbidity burden

Modern quadruple therapy for HF with reduced ejection fraction (HFrEF) extends life expectancy by several years, particularly when implemented early and at scale.^20,21^ Non-CV death can also act as a competing risk, complicating interpretation when morbidity is assessed only as time to first hospitalization. As survival improves, patients spend more time at risk of non-fatal WHF events, as reported in recent registries.^22,23^ Additionally, there is a substantial proportion of WHF episodes treated in ambulatory settings and not limited to hospital care. Ambulatory presentations of WHF are common, and among these, treatment with oral diuretics is significantly more frequent than intravenous therapy. For example, in the VICTOR trial, at least 10 times as many patients with ambulatory HF events received treatment with oral diuretic intensification as IV diuretics.^24^ Thereby, the traditional combined endpoint of time to first of CV death or HF hospitalization loses biological pertinence mainly because it does not fully capture the long-term risk of cumulative HF morbidity burden that occurs during mid and long-term follow-up.^25^ A very simple example illustrates this statement. Two patients may have the same time free of the first HF hospitalization. Yet, one might have lived substantially longer with fewer recurrent events and better functional status. The other one may have experienced a greater number of total WHF episodes treated in an ambulatory setting, representing a higher morbidity burden not captured with the traditional time to first combined endpoint of death or first HF hospitalization.

Analyses from more recent HF trials, which account for the total HF event burden rather than only first events, show that many therapies yield benefits that are similar to, or even exceed, those suggested by the traditional combined endpoint. For instance, in PARADIGM-HF, sacubitril/valsartan reduced the time-to-first composite of CV death or first HF hospitalization by 20%, while recurrent-event modelling showed a 23% lower rate of total (first and recurrent) HF hospitalizations. In EMPEROR-Reduced, empagliflozin lowered the time-to-first primary composite of CV death or first HF hospitalization by 25% but reduced total HF hospitalizations by 30%. Similarly, in DELIVER, dapagliflozin reduced the time-to-first composite of WHF or CV death by 18%, whereas the rate ratio for total (first and recurrent) WHF events plus CV death was reduced by 23%.^9,26–29^ More recently, the VICTOR trial showed a neutral effect of vericiguat regarding the primary endpoint of time to first of CV death/HF hospitalization; however, the effect was positive when including all HF hospitalizations.^30,31^

Thus, there are data suggesting that the reliance on first HF hospitalization as the sole morbidity component risks underestimating morbidity burden and the clinical benefit of many treatment strategies.^24^ In addition, the consistency of estimated treatment effect is maintained with broader definitions that include ambulatory intravenous or oral diuretic intensification. However, recurrent-event approaches are not universally more efficient: in many HF trials, only a minority of participants experience multiple events, and power gains depend on risk heterogeneity and low rates of treatment discontinuation after a first event.^32^

Mortality is also a competing risk for WHF, and the balance of CV versus non-CV death varies by HF phenotype and trial era. For example, in PARADIGM-HF (HFrEF),^9^ CV death occurred in 13.3% vs 16.5% and all-cause death in 17.0% vs 19.8%, indicating that CV death accounted for most deaths in this population. In contrast, in PARAGON-HF (HFpEF),^33^ among 691 deaths, 416 (60%) were CV, 220 (32%) were non-CV, and 55 (8%) were unknown, illustrating a larger competing non-CV death burden. These data explain why all-cause death is the most objective mortality anchor, whereas CV death can add mechanistic specificity but requires a prespecified definition and consistent adjudication to remain interpretable across populations and eras.

### Heterogeneity and context dependence of HF hospitalization

Although HF hospitalization is commonly treated as a relatively objective, bias-free endpoint, it is in fact a composite of heterogeneous clinician and system-level decisions. The decision to admit an individual patient depends not only on HF severity but also on subjective physician judgment, patient demographic characteristics, such as age, sex, and socioeconomic status, patients’ preferences, geography (local practices), social/familial support, reimbursement policies, hospital bed capacity, and the availability of alternative care pathways, such as HF clinics and hospital-at-home programmes.^34^

International analyses show striking intercountry variability in HF hospitalization rates that is not fully explained by patient characteristics.^35^ This system dependence is illustrated by a recent study published in NEJM Catalyst regarding the US health system. In a cohort of 3233 emergency department (ED) visits for HF, 88.7% resulted in inpatient admission. Notably, 96.0% of these admissions were deemed ‘potentially avoidable’ under CMS Prevention Quality Indicators.^36^

Moreover, external non-cardiac triggers such as air pollution or respiratory infections can precipitate WHF and admission, further decoupling ‘HF hospitalization’ from the underlying WHF episode and its severity.^37^ Recognizing this reality, contemporary consensus frameworks have proposed standardized definitions for HF events that emphasize objective signs and therapies rather than location of care.^34,38^ Yet adoption of these definitions in pivotal phase 3 trials remains incomplete. As long as ‘hospitalization for HF’ is used without fully standardized criteria, it will remain a relatively context-dependent endpoint.

### WHF care across ambulatory settings

Contemporary HF care often emphasizes decisively towards early detection and ambulatory management of WHF, mainly driven by congestion.^39,40^ Dedicated HF clinics and multidisciplinary programmes reduce HF hospitalization and all-cause mortality compared with usual care and are now recommended as a core component of HF management.^24,41^ Structured discharge planning and follow-up pathways, together with home-based and remote monitoring strategies, are commonly integrated into routine HF care and offer the potential for detecting congestion early and managing WHF in ambulatory settings.^15,42^

Multicomponent HF management programmes and structured discharge planning have demonstrated mixed effects on readmissions and clinical outcomes across settings and populations.^43–46^ Remote monitoring of implanted devices and telemetric physiological signals may facilitate earlier identification congestion and arrhythmia-related deterioration early and can support ambulatory follow-up and timely clinical reassessment.^47^

Additionally, outpatient tools such as lung ultrasound, inferior vena cava imaging, and biomarker-guided diuretic titration have been shown to reduce WHF and HF admissions by enabling targeted intensification of therapy in clinic or day-hospital settings.^48,49^

Care delivery across inpatient wards to specialized ambulatory environments has two major clinical implications. First, lower HF hospitalization rates observed in some registries and trials may reflect differences in where and how WHF is treated in outpatient settings rather than a reduction in WHF incidence. Second, a hospitalization-based endpoint preferentially captures WHF in systems that still rely heavily on inpatient care, and systematically underestimates morbidity in health systems that have successfully implemented ambulatory pathways.

### Reduced statistical power: lessons from vericiguat

From a theoretical point of view, accounting for only the first morbidity event and including only a subset of those hospitalized reduces the statistical power, increasing the odds of type II error. The limitations of HF hospitalization as a primary endpoint become concrete when examining recent VICTOR and some statin trials in HF. These limitations are compounded by declining baseline rates of death and HF hospitalization in contemporary HF trials, which necessitates larger sample sizes and longer follow-up to detect treatment effects.^17^

#### The example of vericiguat

In VICTORIA, vericiguat reduced the conventional time-to-first composite of CV death or first HF hospitalization in high-risk patients with HFrEF with recent WHF, driven primarily by fewer first HF admissions.^50^ However, the effect size was modest (10% reduction). Post hoc analyses of this trial suggested that when treatment effect was assessed beyond the first event, including total HF events and biomarker trajectories, the signal was more pronounced.^51^

In contrast, the VICTOR trial examined a more stable ambulatory HFrEF population without recent WHF. In that setting, vericiguat was neutral for the conventional time-to-first composite of death or HF hospitalization [hazard ratio (HR) 0.93; 95% confidence interval (CI) 0.83–1.04; *P* = .22].^31^ However, in a secondary analysis using an expanded WHF endpoint that captured outpatient episodes requiring oral diuretic initiation or intensification together with urgent and hospitalized HF events, vericiguat reduced the composite of overall WHF and death by 10% (HR 0.90; 95% CI 0.82–0.98; *P* = .016).^30^ Sensitivity analyses showed that this treatment effect remained consistent even when oral diuretic intensification events were restricted to dose increments of ≥50%.^52^ Taken together, these analyses illustrate more explicitly that treatment effects may appear attenuated when only the first hospitalized HF event is counted, whereas they may become more apparent when total or recurrent HF burden and ambulatory WHF events are incorporated.

## Reasons to move forward: outpatient WHF events are frequent and prognostically crucial

Several studies have highlighted that outpatient WHF episodes portend a meaningful increase in the risk of adverse clinical events albeit generally lower than the risk following an HF hospitalization. In BIOSTAT-CHF, ambulatory patients had high event rates (death or HF hospitalization: 18.5 per 100 person-years), and high-risk outpatients had event rates overlapping those of inpatients.^53^ An analysis of over 100 000 patients within an integrated health system and found that emergency department and outpatient encounters accounted for approximately half of all WHF episodes. Crucially, the study reported that the 30-day risk of a subsequent hospitalization for WHF following an index outpatient encounter was 8.2%, falling within a relatively narrow range of the 12.4% risk observed following an index hospitalization. Consequently, the authors concluded that outpatient WHF represents a sentinel event in the natural history of the disease, carrying a poor short-term prognosis although lower than that following inpatient WHF events.^23^

**Figure 2:**
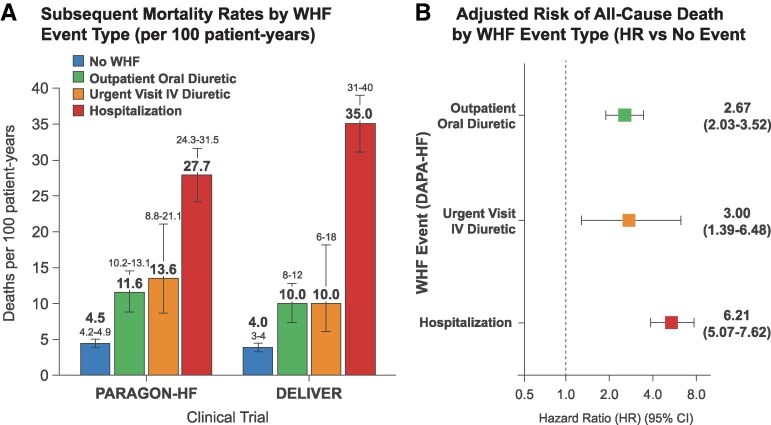
Prognostic impact of ambulatory vs hospitalized WHF events. (A) Subsequent all-cause mortality rates (per 100 patient-years) stratified by the setting of the prior WHF event in the PARAGON-HF and DELIVER trials. (B) Adjusted HR for all-cause death following specific WHF events in the DAPA-HF trial, compared with patients with no preceding event. Error bars indicate 95% confidence intervals. Abbreviations: HF, heart failure; HR, hazard ratio; IV, intravenous; WHF, worsening heart failure

Evidence from randomized clinical trials also suggest that ambulatory HF events are an important endpoint with prognostic relevance.^24^ In recent HF with preserved ejection fraction and HFrEF trials, outpatient WHF has proved to be prognostically relevant: for example, in PARAGON-HF, urgent ambulatory HF visits requiring intravenous diuretics (7.5% of first WHF events) were followed by markedly higher subsequent mortality (10.1 vs 4.0 deaths per 100 patient-years in those without WHF).^54^ In DAPA-HF, the adjusted risk of all-cause death (vs no worsening event) after an outpatient worsening episode was HR 2.67 (95% CI 2.03–3.52), after an urgent HF visit was HR 3.00 (95% CI 1.39–6.48), and after an HF hospitalization was HR 6.21 (95% CI 5.07–7.62);^55^ In EMPEROR-Preserved outpatient, diuretic intensification was frequent and was reduced with empagliflozin [482 vs 610; HR 0.76 (95% CI 0.67–0.86); *P* < .0001).^56^ Also, in DELIVER, intensification of oral diuretic therapy in ambulatory settings identified a high risk subgroup with subsequent mortality rates of 10 (8–12) per 100 patient-years versus 4 (3–4) per 100 patient-years in those without WHF, similar to rates after an urgent HF visit [10 (6–18) per 100 patient-years] and lower than after HF hospitalization [35 (31–40) per 100 patient-years]; inclusion of outpatient diuretic intensification increased first events from 1122 to 1731 (+54%), and dapagliflozin reduced the expanded composite (CV death, HF hospitalization, urgent HF visit, or outpatient oral diuretic intensification) [HR 0.76 (95% CI 0.69–0.84)], consistent with the primary DELIVER composite of worsening HF or CV death [HR 0.82 (95% CI 0.73–0.92)].^27,57^ These data are summarized in *Figure 2*.

These observations have been codified in contemporary conceptual frameworks that define WHF as requiring escalated decongestive therapy, along with characteristic symptoms and signs, irrespective of care setting.^19,58^ Excluding outpatient WHF from trial endpoints has at least three consequences that are directly addressed by a WHF-centred framework. It underestimates the true burden of disease, discards frequent and highly informative events that can substantially increase statistical power, and embeds systematic bias in favour of less developed and organized health care systems.

## Towards a modern, standardized, and comprehensive WHF endpoint

We propose a new operational definition of WHF that accounts for contemporary care pathways and captures clinically meaningful worsening across inpatient and ambulatory settings.

### Anchoring on consensus-based standardized definitions

Published consensus frameworks provide a detailed framework for standardized definitions of HF events across the spectrum of acute and chronic HF.^38^ For WHF, these frameworks are objective evidence of congestion or low output, a requirement for HF-specific therapies, and careful adjudication of competing conditions such as acute coronary syndromes. Importantly, WHF events may occur and be managed in ambulatory or emergency settings, not only inpatient admissions.

### Proposed adjudicated WHF event definition

A standardized WHF definition, informed by published consensus definitions, could serve as the core morbidity component of composite endpoints in future HF trials (*Table 2*, *Graphical abstract*).

**Table 2:** Proposed standardized definition of worsening heart failure (WHF) events

Domain	Subdomain	Criteria for WHF adjudication
Domain 1. Clinical syndrome compatible with WHF	Symptoms	New or worsening HF symptoms compared with the most recent clinically stable status, with at least one of: dyspnoea at rest or with minimal exertion new or worsened orthopnea, bendopnea, or paroxysmal nocturnal dyspnoea rapid increase in fatigue loss of exercise tolerance.
	Signs of congestion or hypoperfusion	At least one objective sign at or near the index contact: elevated JVP above the clavicle at 45° new or worsened peripheral oedema ≥1+ vs prior visit pulmonary rales beyond lung bases ascites or increasing abdominal girth unintentional weight gain suggestive of fluid retention (e.g. ≥1.0–1.4 kg in 24 h or ≥2.3 kg in 7 days). cool extremities narrow pulse pressure
Domain 2. Objective corroboration by biomarkers or device data	Natriuretic peptides relative change	Serial BNP or NT-proBNP: a relative increase in NT-proBNP (or BNP) above an individualized clinically stable (‘dry’) reference value that exceeds expected analytic/biologic variation (e.g. ≈≥30% short-term), interpreted in the context of HF phenotype (including HFpEF), atrial fibrillation, and obesity and value above a prespecified high-risk threshold for the enrolled HF phenotype and care setting.
	Natriuretic peptides absolute threshold	When no stable baseline is available: single BNP or NT-proBNP value above a protocol-defined high-risk threshold, chosen *a priori* from guideline and trial cut-offs for the relevant HF phenotype and care setting.
	Device or imaging congestion metrics	Validated congestion markers can substitute NP in this domain: sustained increase in mean pulmonary artery pressure of about ≥3 mmHg above individual reference or into a protocol-defined upper target range or protocol-defined worsening of lung ultrasound B line burden or other validated congestion indices.
Domain 3. HF-specific therapeutic intensification attributable to the event	HF-specific oral diuretic intensification	Clear escalation of HF directed oral diuretic therapy attributed to congestion: >50% (≥1.5-fold) increase in chronic loop diuretic daily dose for at least 1 week, or any diuretic up titration in cases of patients on high-diuretic doses (furosemide equivalent doses ≥120 mg/day) or addition of a second diuretic class (e.g. thiazide type agent or acetazolamide) with the explicit aim of decongestion.
	HF-specific IV therapy or advanced decongestion	Use of parenteral HF therapies or extracorporeal fluid removal for HF in any care setting: IV loop diuretics IV vasodilators or inotropes initiation of ultrafiltration or other mechanical fluid removal; initiation or escalation of temporary mechanical circulatory support for HF related low output.

Definite vs probable WHF classification. Definite WHF: Domains symptoms, signs, natriuretic peptides or device/imaging, and HF-specific treatment escalation all fulfilled. Probable WHF: clinical domains (symptoms and treatment escalation) fulfilled but biomarker or device data missing in the event window, to be used only in prespecified sensitivity analyses and not in the primary WHF endpoint.

Core domains include clinical worsening, objective evidence (e.g. biomarkers), and treatment intensification for event classification.

#### Domain 1. Clinical syndrome compatible with WHF

Domain 1 establishes the clinical plausibility that the episode represents WHF. It requires new or worsening HF symptoms compared with the most recent clinically stable status, together with at least one objective sign of congestion or hypoperfusion at or near the index contact. The purpose of this domain is to ensure that events are anchored in clinically meaningful worsening rather than transient symptom fluctuation or non-HF diagnoses. Operational examples of qualifying symptoms and signs, including bendopnea, weight gain suggestive of fluid retention,^59^ and bedside evidence of congestion or hypoperfusion, are detailed in *Table 2*.

#### Domain 2. Objective corroboration by biomarkers or device data

Domain 2 provides objective corroboration that the clinical syndrome reflects worsening HF pathophysiology. Whenever feasible, the event should be supported by protocol-defined biomarker evidence of congestion, preferably serial BNP or NT-proBNP interpreted relative to an individualized clinically stable (‘dry’) reference value and requiring a rise that exceeds expected analytic/biologic variation, together with a value above a prespecified high-risk threshold appropriate for the HF phenotype and care setting. A fixed relative rise of approximately 30% in NT-proBNP or BNP should be regarded as a pragmatic anchor rather than a universally validated threshold, and interpretation should take into account HF phenotype (including HFpEF), atrial fibrillation, and obesity. This approach is supported by studies of biological variation in chronic HF, where intraindividual coefficients of variation for NT-proBNP are around 20% and reference change values are around 60%, and by clinical series showing serial change values of 23% to 30% in carefully stabilized patients.^60–62^ When no stable baseline value is available, a single BNP or NT-proBNP value above a protocol-defined high-risk threshold chosen *a priori* from guideline- and trial-based cut-offs for the relevant HF phenotype and care setting may be used.

Validated congestion markers may also substitute natriuretic peptide criteria in this domain, including a sustained increase in mean pulmonary artery pressure of approximately ≥3 mmHg above the individual reference or target value, protocol-defined worsening of lung ultrasound B-line burden, or other validated congestion indices. This domain is intended to improve specificity, strengthen adjudication consistency across sites and care settings, and support sensitivity analyses distinguishing definite from probable WHF when objective data are unavailable within the event window. If natriuretic peptide measurements and device data are not available within the event window, but Domains 1 and 3 are fully satisfied, the episode may be classified as probable WHF for prespecified sensitivity analyses. Only definite WHF events meeting Domain 2 should contribute to the primary WHF endpoint. Operational thresholds, acceptable data sources, and substitution rules are detailed in *Table 2*.

#### Domain 3. HF-specific therapeutic intensification attributable to the event

Domain 3 confirms that the episode prompted HF-specific therapeutic intensification attributable to congestion or HF-related clinical deterioration. Consistent with consensus frameworks for standardized HF endpoint definitions,^38^ the treating clinician must judge HF as the primary driver of worsening, and the medical record must document escalation of HF-directed therapy across care settings. This domain deliberately focuses on treatment intensification rather than location of care, acknowledging that clinically important WHF may be managed in outpatient, HF clinic, emergency, observation, day-hospital, hospital-at-home, or inpatient settings. Qualifying interventions include clear escalation of oral diuretic therapy, such as a >50% (≥1.5-fold) increase in chronic loop-diuretic dose sustained for at least 1 week, any up-titration in patients already receiving high baseline diuretic doses (furosemide-equivalent dose ≥120 mg/day), or addition of a second diuretic class with the explicit aim of decongestion, as well as parenteral HF therapies or advanced decongestion strategies, including IV loop diuretics, IV vasodilators or inotropes, extracorporeal fluid removal, or temporary mechanical circulatory support for HF-related low output. Contemporary trials and registries have used outpatient oral diuretic intensification to operationalize WHF and identify a high-risk trajectory, with event rates after intensification approaching those seen after urgent HF visits or hospitalization.^38,52,57^ Escalations driven predominantly by non-HF conditions, for example isolated pneumonia without clear HF worsening consistent with WHF, primary renal failure, or sepsis, should not be adjudicated as WHF even if diuretics are modified. The operational definitions of qualifying treatment changes, including oral and parenteral strategies and documentation standards, are provided in *Table 2*.

#### Event counting rules

- WHF events are counted irrespective of care setting and are labelled as hospitalized or non-hospitalized for descriptive purposes only.
- Multiple health care contacts related to the same clinical deterioration are considered one WHF episode if they occur within a 30-day window without clear documentation of resolution.
- A window of up to 48 h after the index contact is allowed to complete biomarker and device measurements required to adjudicate the event.
- A new WHF event can be counted only after at least 30 days without WHF criteria and without ongoing therapeutic intensification for the previous episode.

Our proposed definition builds directly on HF-ARC recommendations for WHF but goes a step further in three key ways: it unifies hospitalized and non-hospitalized events into a single three-domain structure, specifies quantitative thresholds for biomarkers and device-based congestion metrics, and distinguishes definite from probable WHF for use in primary and sensitivity analyses. These features make the definition immediately operational as a primary trial endpoint component in chronic HF, rather than solely as a conceptual classification framework.

### A hierarchical primary endpoint for HF trials

Because mortality remains the most important outcome in HF, hierarchical composite endpoints could be considered as primary endpoints in chronic HF trials, ordered by clinical priority rather than restricted to time-to-first hospitalization. By capturing outpatient WHF and recurrent events, such endpoints can increase event counts and potentially improve statistical power when these events are frequent.

#### First level: mortality

The top-ranked component is time to all-cause death. Cause-specific mortality (e.g. CV death) can be defined and, when appropriate, adjudicated using prespecified standardized criteria^38^ and reported as a key secondary endpoint, recognizing that proportions and competing risks vary by population, region, and trial era. For therapies not expected to modify mortality, trials may prioritize morbidity and patient-reported outcomes while retaining death as a critical safety and interpretability anchor.

#### Second level: total adjudicated WHF events across all care settings

The second level is the total burden of definite WHF events, as defined in Section 4.2, including both first and recurrent episodes and counted irrespective of care setting. This component incorporates hospitalized WHF, as well as WHF treated in emergency departments, observation units, HF clinics, day hospitals, or hospital-at-home programmes, provided that all objective criteria are met. Within this morbidity tier, trials may prespecify additional prioritization by care setting or acuity (e.g. ranking HF hospitalization or urgent IV-treated events above outpatient oral diuretic intensification).

#### Third level: patient-centred functional and health status outcomes

Validated patient-reported outcome measures, including disease-specific instruments such as the Kansas City Cardiomyopathy Questionnaire and the Minnesota Living with Heart Failure Questionnaire, provide complementary information on how patients feel and function (64–66) [4] and have among the best-documented psychometric performance in.^63^ Generic health status instruments (e.g. EQ-5D, SF-36, SF-12, PROMIS Global Health and PROMIS Physical Function) may further support cross-disease comparisons and health economic analyses. Objective measures of function with supporting validation in HF include cardiopulmonary exercise testing (peak VO_2_ and VE/VCO_2_ slope) and performance-based tests such as 6-minute walk distance, gait speed, and the Short Physical Performance Battery, selected based on trial feasibility and HF phenotype. Interpretation of change should be anchored to clinically meaningful change in the target population and prespecified for the trial context.

### Primary analysis strategy

Primary analysis strategies need to be prespecified and aligned with the chosen endpoint hierarchy. Contemporary trials have already incorporated outpatient WHF events, including oral diuretic intensification, within prioritized composite endpoints (e.g. ELEVATE-HFpEF; NCT06678841, and SUMMIT.^64^)

## Implications for trial design, practice, and regulation

### Trial design and conduct

Implementing a WHF-centred hierarchical endpoint has concrete implications for trial design and conduct in at least four areas:

- **Systematic capture of WHF events across settings:** Trials may prespecify how WHF events will be identified and captured in each care setting, including inpatient wards, emergency departments, HF clinics, day hospitals, hospital-at-home programmes, and remote monitoring platforms. This typically requires standardized WHF reporting templates in HF clinics, linkage to electronic health records for unscheduled visits where feasible (when longitudinal EHR linkage is unavailable, structured patient contact and systematic retrieval of external records can be used) and explicit procedures to translate remote monitoring alerts into clinically verified and documented WHF events (e.g. prespecified alert thresholds, confirmation workflows, and standardized documentation templates).
- **Scheduled biomarker assessment and device data integration:** Protocols may include planned NT-proBNP assessments at baseline and at regular intervals, with clear rules for what constitutes a meaningful change from a stable baseline. In participants with implanted or wearable devices, automated transmission and, when feasible, centralized access of key physiologic signals, such as pulmonary artery pressures or congestion indices, should be organized, so they can be reviewed alongside clinical data when adjudicating suspected WHF events. In many jurisdictions, identifiable device data may need to remain within clinical or vendor platforms; trials need to prespecify data governance, privacy protections, and responsibilities for clinical action on safety alerts.
- **Centralized adjudication using a standardized WHF definition:** Endpoint adjudication committees should apply a standardized WHF definition to all suspected WHF events, irrespective of care setting or geographic region. Trial documents should specify source data requirements for adjudication in different contexts, for example minimum clinical, biomarker, and device data elements for ambulatory WHF, and provide training and calibration exercises to reduce variability between adjudicators and regions.
- **Recurrent event and hierarchical analyses:** Statistical analysis plans should be prespecified, aligned with the chosen endpoint hierarchy, and transparent about how recurrent WHF episodes, competing risks, and missing health status data are handled. Sensitivity analyses using conventional time-to-first event approaches can aid interpretability and comparability across trials.

### Clinical practice and health system evaluation

A shift towards standardized WHF endpoints has implications beyond clinical trials. Health systems increasingly benchmark performance using HF readmission metrics. Adopting standardized WHF definitions that include outpatient WHF events would provide a more accurate picture of system performance and may incentivize high-quality ambulatory HF care rather than penalizing hospitals that manage more WHF events in structured outpatient pathways.

### Regulatory and guideline perspectives

Regulatory agencies and guideline writing committees have already signalled a willingness to move beyond simple composites of CV death or first HF hospitalization. Recent evaluations of sodium-glucose cotransporter 2 inhibitors, angiotensin receptor neprilysin inhibitors, and vericiguat have explicitly considered broader frameworks that incorporate total HF events, WHF episodes, and patient-centred outcomes when interpreting treatment effects and guideline recommendations.^50,65,66^ Similarly, the SUMMIT trial incorporated outpatient oral diuretic intensification within its primary worsening HF composite endpoint.

What is still lacking is a unified, standardized approach to defining WHF and structuring composite endpoints across pivotal trials. Formal endorsement of consensus-based standardized WHF definitions and appropriately prespecified hierarchical or prioritized endpoint frameworks by major regulatory and professional societies could accelerate convergence in trial design, facilitate cross-trial comparability, and reduce ambiguity when therapies show discordant effects on mortality, HF hospitalization, and health status. Such endorsement would also provide clear methodological guidance for therapies whose primary benefit is the reduction of recurrent WHF and stabilization of patient-reported outcomes rather than large early effects on mortality alone.

## Conclusions

The composite endpoint of CV death or first HF hospitalization has served the field of HF well, but in an era of improved survival, ambulatory HF programmes, and digitally supported care, it is no longer sufficient as the default measure of therapeutic efficacy. We propose that future HF trials adopt a standardized, WHF centred hierarchical endpoint in which all-cause mortality remains the highest priority, with CV death assessed as a secondary mortality outcome when prespecified and adjudicated, and total WHF events across all care settings form the core morbidity component, with patient centred outcomes incorporated when they are a key therapeutic target. Such an endpoint is feasible to implement with contemporary data systems and is better aligned with the trajectories of HF that matter to patients, clinicians, and health systems.

## Declarations

### Data Availability

No new data were generated or analysed in support of this research.

### Use of Artificial Intelligence

The authors used ChatGPT to assist with language editing and drafting support, and Nano Banana Pro to assist with figure design. All AI-generated outputs were critically reviewed, verified, and edited by the authors, who take full responsibility for the final content of the manuscript and figures.
